# Metformin in non-diabetic patients with autosomal dominant polycystic kidney disease: a systematic review and meta-analysis of randomized controlled trials

**DOI:** 10.1186/s12882-025-04575-5

**Published:** 2025-11-18

**Authors:** Vitor Almeida, Lucas Maciel, Ana Beatriz Valverde Ramos, Carla Sousa, Maria Ferraz, Luisalice Afonso, Paula Dibo, Ivana Nunes

**Affiliations:** 1 Federal University of Catalão, Catalão, Goiás Brazil; 2 General Hospital of Goiânia, Goiânia Goiás, Brazil; 3 Pontifical Catholic University of Paraná, Paraná Curitiba, Brazil; 4 Jean Piaget University at Angola, Luanda, Angola; 5 Vila Velha University, Vila Velha, Espírito Santo Brazil; 6 Federal University of Cariri, Ceará, Brazil; 7 Division of General Internal Medicine, Department of Medicine, Emory University, Atlanta, Ga USA; 8 Division of Nephrology, Department of Internal Medicine, General Hospital of Goiânia, Goiás, Brazil

**Keywords:** Metformin, Autosomal dominant polycystic kidney disease, Kidney function rate decline, Height-adjusted total kidney volume

## Abstract

**Introduction:**

Autosomal dominant polycystic kidney disease (ADPKD), which is caused mainly by mutations in the PKD1 or PKD2 genes, is a genetic disorder characterized by the growth of cysts and decreased kidney function. There is limited evidence on pharmacological interventions capable of slowing down disease progression in non-diabetic patients. Metformin, widely used to treat type 2 diabetes, has shown potential nephroprotective effects through activation of AMPK and inhibition of the mTOR pathway.

**Methods:**

PubMed, Embase and Cochrane databases were searched for randomized clinical trials (RCTs) comparing metformin versus placebo or standard care in non-diabetic patients with ADPKD. Standardized mean differences (SMDs) and risk ratios (RRs) with 95% confidence intervals (CIs) were calculated via random-effects model. Heterogeneity was assessed using the I2 test. Statistical analyses were performed using Review Manager, version 5.4, and R Software, version 4.4.2.

**Results:**

Four RCTs were included, comprising 213 patients. Average follow-up ranged from 0.15 to 2 years. No significant differences were observed in the decline of kidney function (SMD: 0.19; 95% CI, −0.08 to 0.46; *p* = 0.17) or in height-adjusted total kidney volume (htTKV) progression (SMD: 0.09; 95% CI, −0.20 to 0.38; *p* = 0.53). Gastrointestinal adverse events were more frequent in the metformin group (RR: 2.93; 95% CI, 1.51 to 5.67; *p* = 0.0014), while the incidence of hypoglycemia did not differ between groups (RR: 1.04; 95% CI, 0.36 to 3.00; *p* = 0.948). The pooled prevalence of tolerability-related discontinuation or dose reduction due to adverse effects was 37.38% (95% CI: 12.15 to 72.04%).

**Conclusion:**

This meta-analysis suggests that metformin does not significantly affect the rate of kidney function decline in non-diabetic patients with ADPKD. Its impact on kidney volume remains uncertain, while gastrointestinal symptoms, although more common, were generally mild. However, interpretation is limited by the small number of trials, modest sample sizes, and relatively short follow-up durations, which reduce the ability to assess long-term outcomes. Larger and longer studies are needed to clarify the potential role of metformin in this population.

**Clinical trial registration:**

Not applicable.

**Registration:**

PROSPERO CRD420251062402.

**Supplementary Information:**

The online version contains supplementary material available at 10.1186/s12882-025-04575-5.

## Introduction

Autosomal Dominant Polycystic Kidney Disease (ADPKD) is a hereditary condition characterized by multiple bilateral kidney cysts and an irreversible decline in kidney function [1, 2]. It is the leading genetic cause of chronic kidney disease (CKD), accounting for 5–10% of all cases of kidney failure. Beyond its clinical burden, ADPKD also imposes a substantial socioeconomic cost, with annual healthcare expenditures exceeding USD 7 billion in the U.S [3–5]. Despite this, few disease-modifying agents have been shown to slow kidney function decline in this population, and current management strategies remain largely supportive. To date, tolvaptan remains the only agent approved by the U.S. Food and Drug Administration (FDA) for the management of ADPKD, whereas octreotide has been evaluated in clinical trials but has not received FDA approval yet. However, the clinical use of tolvaptan is constrained by its aquaretic effects, risk of hepatotoxicity, and high cost, while octreotide has demonstrated inconsistent efficacy in preserving kidney function [6, 7].

Metformin, traditionally used in type 2 diabetes, has shown promising results in preserving kidney function and has emerged as a potential therapeutic option due to its pleiotropic effects [8, 9]. Preclinical studies demonstrate that metformin inhibits mitochondrial complex I, increasing the AMP/ATP ratio and activating AMP-activated protein kinase (AMPK). This activation suppresses mTORC1 signaling, reduces CFTR-mediated chloride secretion, and limits tubular cell proliferation and cyst fluid secretion, all of which contribute to cystogenesis in ADPKD [10, 11]. Experimental studies also indicate a synergistic effect with the glycolysis inhibitor 2-deoxy-D-glucose (2DG), further supporting the rationale for targeting cellular energetics in this disease [12, 13].

Beyond its metabolic effects, metformin also exerts anti-inflammatory and antifibrotic effects and may improve vascular function, all of which are thought to contribute to CKD progression [14, 15]. These properties make metformin a biologically plausible, low-cost, and widely available candidate for the management of ADPKD, particularly among non-diabetic patients. However, existing clinical studies are limited by small sample sizes and heterogeneity, underscoring the need for further research to establish more definitive conclusions.

Prior meta-analyses [16, 17] showed that metformin had a favorable safety profile in patients with ADPKD, but the scope and included studies differ from the present work. Our analysis incorporates additional RCTs and focuses specifically on non-diabetic patients, aiming to provide updated and complementary evidence.

## Materials and methods

This systematic review and meta-analysis was registered in the International Prospective Register of Systematic Reviews (PROSPERO) database under registration number CRD420251062402 and conducted in accordance with the Cochrane Handbook for Systematic Reviews of Interventions and Preferred Reporting Items for Systematic Reviews and Meta-Analyses (PRISMA) 2020 guidelines [18]. No ethical approval was needed, as no primary patient data were used.

### Eligibility criteria

We included studies if they met all the following criteria: (1) randomized controlled trials (RCTs), including both parallel and crossover designs, (2) involving non-diabetic adult patients with autosomal dominant polycystic kidney disease (ADPKD); comparing metformin with placebo or standard care. We excluded: (1) non-randomized studies; (2) studies involving pediatric populations; (3) or those lacking control group or relevant outcome data. No restrictions were applied regarding language or year of publication. Patients with impaired glucose tolerance (non-diabetic) were eligible for inclusion, whereas those with diabetes were excluded. We focused on adult patients only to ensure consistency and clinical relevance, as most evidence on metformin in ADPKD is derived from adults. Pediatric studies are scarce, and their inclusion would have added variability without sufficient data for reliable conclusions.

### Search strategy and data extraction

**Fig. 1:**
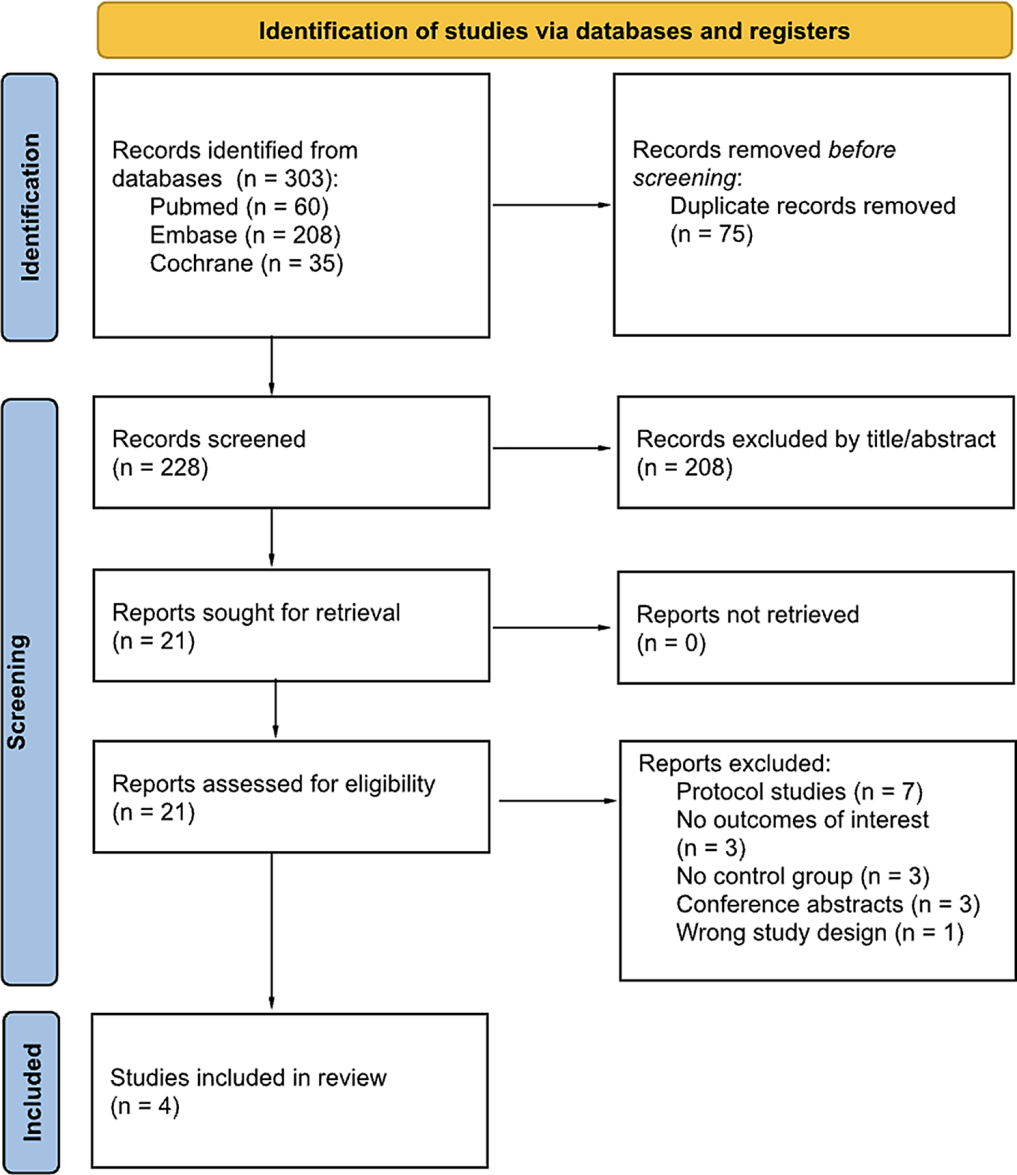
PRISMA 2020 flow diagram of the study selection process. After screening 303 records and reviewing 21 full-text articles, four randomized controlled trials were included in the meta-analysis. Source: Page MJ et al., BMJ 2021;372:n71. doi: 10.1136/bmj.n71. This figure is adapted under the CC BY 4.0 license

A comprehensive literature search was conducted in PubMed, Embase, and the Cochrane Central Register of Controlled Trials from inception to April 20, 2025. Figure 1 presents the PRISMA flow diagram used for the purpose of this study; the search terms used included combinations of “autosomal dominant polycystic kidney disease” and “metformin”. The complete search strategy is provided in the Supplementary Material. In addition, the references of selected articles were manually reviewed to identify any additional studies that might have been missed by the electronic searches. Two reviewers (V.P.A and A.B.V) independently screened titles, abstracts, and full texts, and extracted relevant data using a piloted Excel form designed to minimize transcription errors. A third reviewer (I.V.N) was consulted to resolve discrepancies. For crossover trials, only data from the clinical treatment period were extracted. After initial extraction, a third author (L.G.M) verified the data for accuracy and consistency.

### Outcomes

The included outcomes were: (1) kidney function rate decline; (2) change of total kidney volume (htTKV); (3) gastrointestinal effects; (4) hypoglycemia and (5) metformin tolerability defined as dose reduction or discontinuation of metformin due to these adverse effects. Efficacy outcomes comprised the rate of eGFR decline and changes in htTKV, while safety outcomes included tolerability, gastrointestinal events, and hypoglycemia.

### Quality assessment

Two authors (V.P.A. and M.F.) independently assessed the quality and risk of bias. Disagreements were resolved with a third author (I.V.N.). The Cochrane Collaboration’s Risk of Bias-2 (RoB-2) tool was used to evaluate the risk of bias in randomized trials. RoB-2 has 5 domains, specifically selection, performance, detection, attrition, and reporting [19].

Quality of evidence for each finding was rated based on criteria established by the Grading of Recommendations Assessment, Development, and Evaluation (GRADE) group [20]. RCTs were considered to be high-quality evidence, which could be downgraded to moderate, low, or very low quality for five reasons (high risk of bias, inconsistent results, indirect evidence, imprecision, and publication bias). Disagreements were settled by consensus.

### Statistical analysis

We performed all analyses as per the Cochrane recommendations [21]. Continuous outcomes were analyzed as standardized mean difference (SMD) with 95% confidence intervals (CIs), and were pooled using the inverse-variance method. Binary outcomes were analyzed using Risk Ratios (RR) with 95% CI. The Mantel–Haenszel method was applied for RR comparisons in binary data. A random-effects model was used in all analyses to account for clinical and methodological heterogeneity. Heterogeneity was assessed using the I^2^ statistic and Cochran’s Q test, with I^2^ > 25% and *p* < 0.10 considered significant. The Wald-type method was used to summarize the Cls. Leave-one-out sensitivity analyses were performed to test robustness of our analyses. All analyses were conducted via Review Manager (RevMan) version 5.4 (Nordic Cochrane Centre, The Cochrane Collaboration, Copenhagen, Denmark) and R Software version 4.4.2 (R Foundation for Statistical Computing).

An exploratory analysis was performed to test the tolerability at metformin use. For that purpose, proportional meta-analyses were reported in percentages (rates), with 95% CI. Initially, Freeman-Tukey double arcsine transformation was used to account for studies with zero events. Given the presence of substantial difference about time of follow-up of one trial [22], a sensitivity analysis was subsequently performed excluding this study. For this secondary analysis, a generalized linear mixed-effects model (GLMM) with logit transformation (PLOGIT) was applied.

## Results

### Study selection and characteristics

As depicted in Fig. 1, our initial search yielded 303 records. After removing duplicate reports and excluding studies on the basis of title and/or abstract, 21 studies underwent full review. Four RCTs met the inclusion criteria and were included. The detailed characteristics of all eligible trials are presented in Table 1. A total of 213 non-diabetic patients with ADPKD were included, of whom 114 (50.5%) used metformin and 112 (49.5%) were in the control group. All studies included in the analysis were reported between 2021 and 2024 and the sample size of the treatment arm of each trial varied from 13 to 49. The duration of follow-up ranged from 1.86 to 24 months. Participants were predominantly female between both metformin vs non-metformin users in all studies. All studies used metformin to treat non-diabetic patients with ADPKD.

**Table 1:** Baseline characteristics of included studies

Study, years	Patients, no. M/C	Follow- up, months	Female sex M/C (%)	Age^†^, M/C years	eGFRcre, M/C (mL/min/1.73 m^2^)	Mayo Class 1A and 1B, M/C (%) *	Mayo Class 1C, 1D and 1E, M/C (%) *
Brosnahan, 2022 [23]	26/25	12	58/68	48/48	68/72	27/36	73/64
Kramers, 2022 [22]	13/13	1.9	54/54	45/45	55/55	31/31	69/69
Perrone, 2021 [8]	49/48	24	77.6/66.7	41.8/42.1	86.1/85.9	44.8/43.7	55.2/56.3
Venkatasubramania, 2024 [24]	26/26	6	53.87/57.69	38.23/36.08	100.11/100.34	76.9/84.6	23.1/15.4

†mean or median; * Mayo Class MIC 1A and 1B were combined into a single composite group, as were Mayo Class MIC 1C, 1D; and 1E; M: metformin users; C: Control group; SD: Standard deviation; eGFR: estimated Glomerular Filtration Rate

### Pooled analysis of all studies

**Fig. 2:**
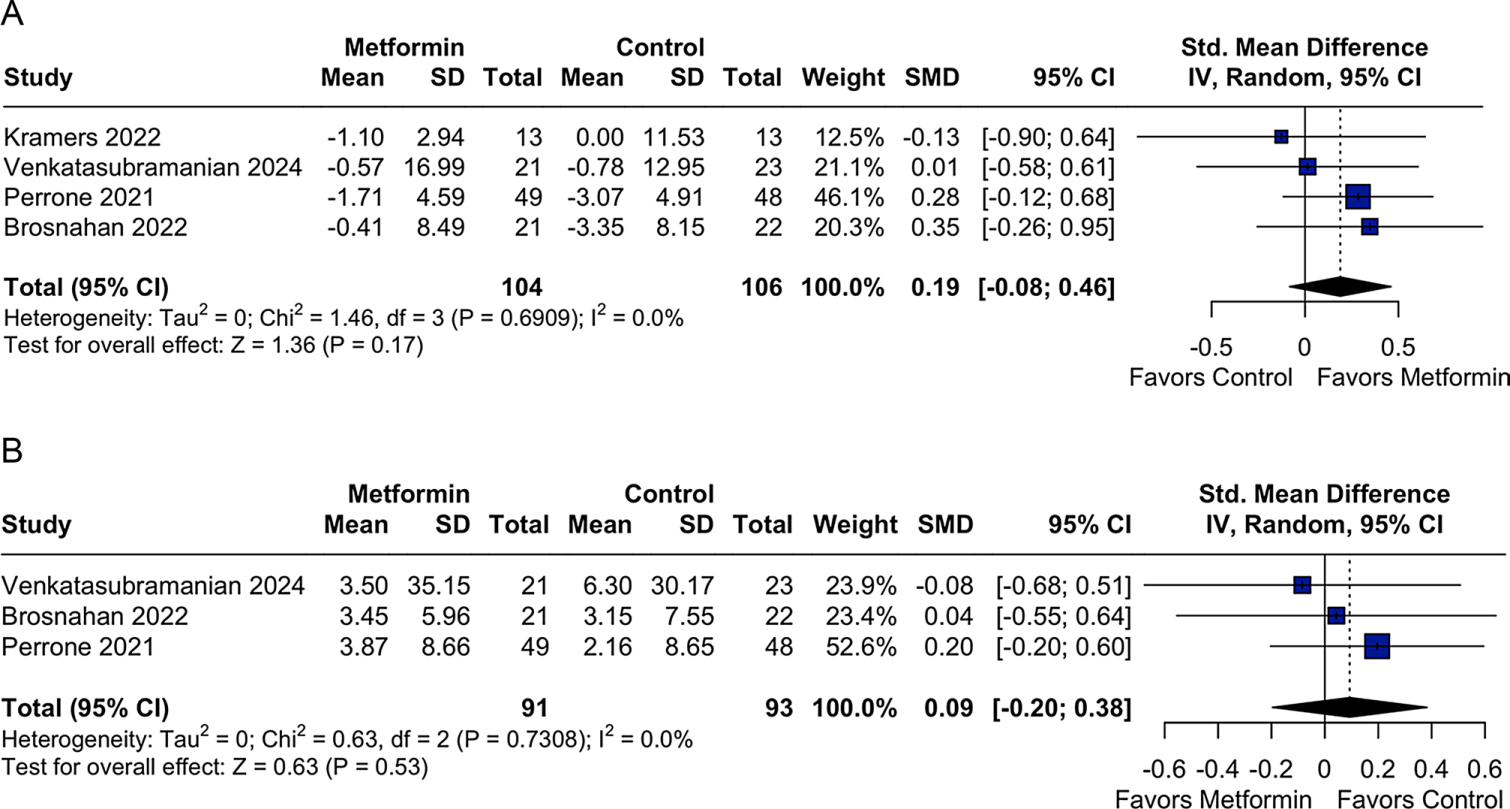
A Forest plot for kidney function rate decline as measured by estimated glomerular filtration rate (eGFR). Legend: patients with autosomal dominant polycystic kidney disease (ADPKD) using metformin showed a similar rate of eGFR decline compared to controls, with no significant difference across trials. CI: confidence interval; smd: standardized mean difference. B Forest plot for changes in height-adjusted total kidney volume (htTKV). Legend: Changes in htTKV were comparable between the metformin and control groups among non-diabetic patients with autosomal dominant polycystic kidney disease (ADPKD). No significant differences were observed across studies. CI: confidence interval; SMD: standardized mean difference

Metformin use was not associated with a significant difference in kidney function, as measured by the decline in eGFR, compared with the control group (SMD 0.19; 95% CI: −0.08 to 0.46; *p* = 0.17; I^2^ = 0%; Fig. 2A). Similarly, no significant difference was observed between groups in the progression of htTKV (SMD 0.09; 95% CI: −0.20 to 0.38; *p* = 0.53; I^2^ = 0%; Fig. 2B).

When the Kramers et al. [22] was excluded from the analysis, due to its short follow-up duration of only 8 weeks, which is likely insufficient to detect meaningful changes in a slowly progressive disease such ADPKD, the pooled analysis comprised three RCTs, including a total of 184 patients. The results showed no statistically significant difference in the rate of eGFR decline between the metformin and control groups (SMD 0.23; 95% CI −0.06 to 0.52; *p* = 0.12; I^2^ = 0%; Supplementary Figure 4). These findings should be interpreted with caution, given the small number of available studies and their relatively short follow-up durations.

In clinical terms, the decline in eGFR reported across the included trials ranged from approximately 0.41 to −3.35 mL/min/1.73 m^2^, while the mean change in htTKV ranged from +11.57 to +102.7 mL/m. These values are consistent with the pooled estimates; however, because of variation in reporting formats across studies, SMDs were used in the meta-analysis to allow direct quantitative comparison across studies, as reported above.

**Fig. 3:**
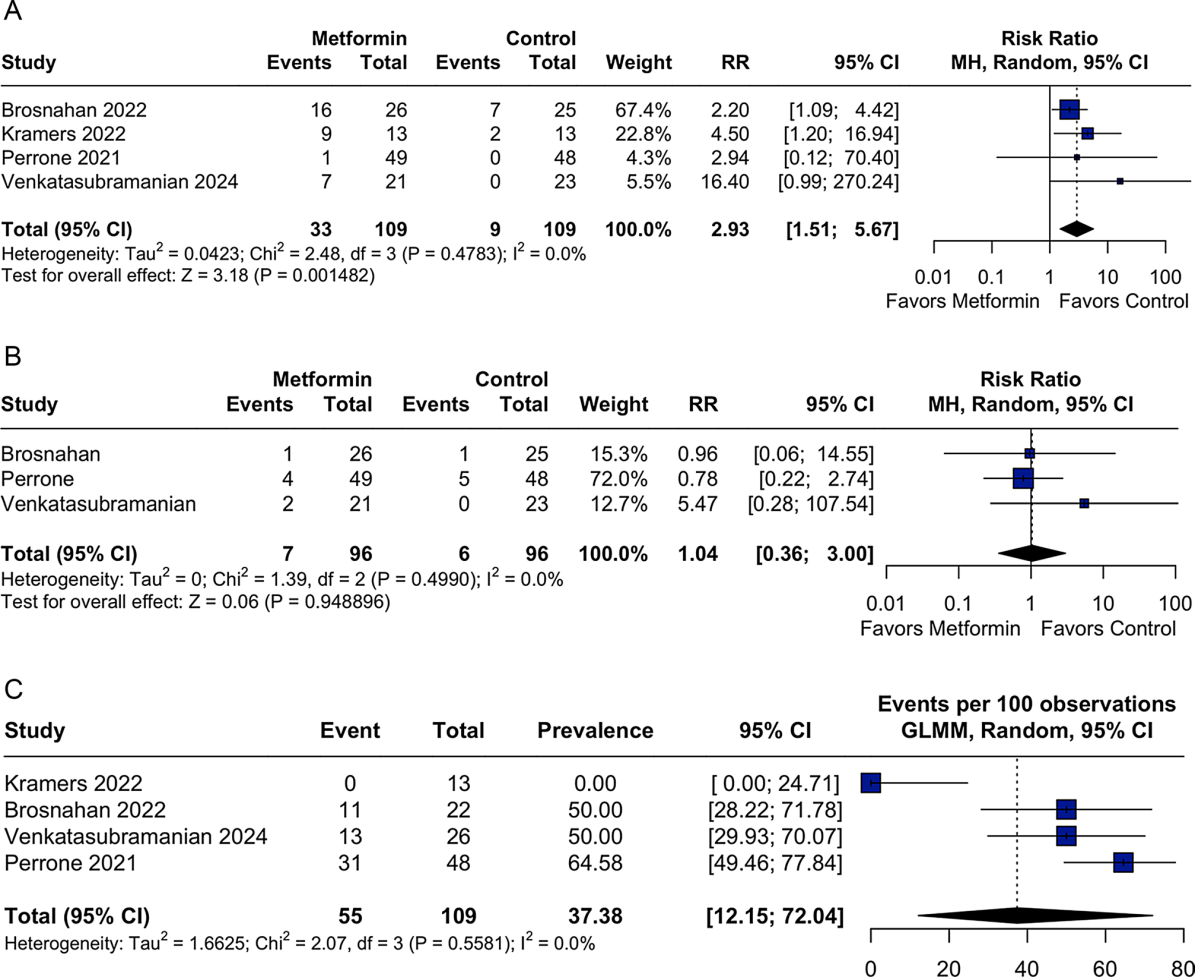
A Forest plot for gastrointestinal adverse events. Legend: gastrointestinal events were significantly more common in patients receiving metformin than in controls. CI: confidence interval; rr: risk ratio. B Forest plot for hypoglycemia. Legend: the incidence of hypoglycemia was similar between the metformin and control groups in non-diabetic patients with ADPKD. No significant difference was observed across studies. CI: confidence interval; rr: risk ratio. C Forest plot for tolerability-related adverse events. Legend: The prevalence of tolerability-related adverse events, defined as dose reduction or discontinuation of metformin due to adverse effects, varied across studies but showed a pooled rate of 37.38%. No significant heterogeneity was observed. CI: confidence interval; GLMM: generalized linear mixed-effects model

Regarding safety, metformin was associated with a significantly higher incidence of gastrointestinal adverse events (RR 2.93; 95% CI: 1.51 to 5.67; *p* = 0.0014; I^2^ = 0%; Fig. 3A). However, the incidence of hypoglycemia did not differ significantly between groups (RR 1.04; 95% CI: 0.36 to 3.00; *p* = 0.948; I^2^ = 0%; Fig. 3B).

The pooled prevalence of tolerability-related adverse events due to discontinuation or dose reduction was 37.38% (95% CI: 12.15 to 72.04; I^2^ = 0%; Fig. 3C). An extra-analysis excluding Kramers et al. [22], which reported no adverse events and had a short follow-up period, yielded a more consistent estimate, with a pooled prevalence of 56.7% (95% CI: 46.71 to 66.18%; I^2^ = 0%; Supplementary Figure 1).

### Quality assessment

Individual appraisals of RCTs are detailed in Supplementary Table 1. The crossover trial by Kramers et al. [22] presented a low risk of bias across all assessed domains, including the randomization process, carryover effects, and outcome measurement, indicating robust internal validity. Among the parallel-group RCTs, only one study demonstrated a consistently low risk of bias across all RoB 2.0 domains [8]. In contrast, Brosnahan et al. [23] and Venkatasubramanian et al. [24] were judged as having “some concerns”, particularly due to issues in the randomization process, missing outcome data, and selective reporting of results.

As presented in Supplementary Figures (2A–D) the funnel plot appeared relatively symmetrical, with effect estimates converging toward the pooled treatment effect as standard errors decreased. Nonetheless, because only four studies were included, the ability to detect publication bias is extremely limited, and no true conclusions can be drawn.

Leave-one-out sensitivity analyses are presented in the Supplementary Figures (3A–E). Overall, the pooled estimates for eGFR decline, htTKV progression, gastrointestinal adverse events, hypoglycemia, and tolerability remained consistent, with only minor changes in heterogeneity depending on the excluded trial, particularly in outcomes influenced by differences in follow-up duration or sample size.

Based on the GRADE assessment presented in Supplementary Table 2, the certainty of evidence in non-diabetic patients ADPKD ranged from moderate to high. No major concerns were identified regarding risk of bias, inconsistency, or publication bias. Efficacy outcomes, including eGFR decline and htTKV progression, were rated as low certainty due to few available trials, a low number of events, and short follow-up. Safety outcomes, including gastrointestinal events and hypoglycemia, were rated as moderate certainty for similar reasons. Overall, these findings indicate that evidence is limited, and further well-designed, larger, and longer-term studies are needed to provide more robust estimates and inform clinical decisions.

## Discussion

This systematic review and meta-analysis evaluated the efficacy and safety of metformin in non-diabetic patients with ADPKD. A total of four RCTs [8, 22–24] comprising 213 patients were included. The main findings were as follows: (1) no significant difference was observed in the decline of eGFR or in the (2) htTKV; (3) gastrointestinal adverse events were significantly more common in the metformin group compared with controls; (4) the risk of hypoglycemia did not differ between groups; and (5) tolerability issues were observed, with dose reduction or discontinuation required in some patients due to gastrointestinal complaints, asthenia, and other adverse effects.

ADPKD is the most common inherited cause of chronic kidney disease and is characterized by progressive cyst growth and kidney enlargement. Although disease-modifying therapies for ADPKD remain limited, metformin has attracted attention because of its antiproliferative properties, mediated via activation of AMPK/mTOR/CFTR pathways involved in cystogenesis [4, 8, 12]. Since few studies have demonstrated ways to slow kidney function decline, the development of novel therapies remains a high clinical priority [23].

In our study, both the metformin and control groups showed declines in kidney function, but the difference between groups was not statistically significant. The absence of a statistically significant effect in our meta-analysis may be explained by a few factors. First, most included trials enrolled patients with relatively preserved kidney function, limiting the ability to detect differences in eGFR decline. Second, the follow-up duration in two studies [22, 24] was relatively short (≤6 months), which may have been insufficient to observe a protective effect of metformin in patients with stable kidney function. Third, the low number of included studies and small sample size may have limited the power to detect differences in eGFR outcomes. Nevertheless, the numerically less pronounced decline in the metformin group may suggest a possible trend toward kidney protection, though this finding should be interpreted with caution.

This aligns with the TAME-PKD trial [8], which evaluated non-diabetic ADPKD patients and reported a numerical signal favoring metformin, although without statistical significance. Similarly, the meta-analysis by Yao et al. [25], which included mixed populations, suggested a significant attenuation of eGFR decline with metformin, without an effect on htTKV. In parallel, the Cochrane review on ADPKD [17] reported a numerical but non-significant signal favoring metformin on eGFR, without an effect on htTKV, while the broader Cochrane review on chronic kidney disease of mixed etiologies [16] suggested a modest attenuation of kidney function decline with metformin, although with low certainty of evidence.

Regarding anatomical progression, htTKV is recognized by both the FDA and European Medicines Agency (EMA) as a prognostic biomarker, and in 2018 the FDA designated total kidney volume (TKV) as a ‘reasonably likely’ surrogate endpoint for disease progression [4, 26, 27]. In our analysis, no significant effect on htTKV was observed. Likewise, Yao et al. [25] and the Cochrane review on interventions in ADPKD [16] found no significant change in htTKV. These findings suggest that any potential benefit from metformin may happen independently of structural kidney changes, at least within the timeframe of current studies.

In terms of safety, gastrointestinal adverse events were significantly more common in the metformin group, consistent with known tolerability issues and prior findings from Yao et al. [25] and Cochrane reviews [16, 17]. Both Brosnahan et al. [23] and the crossover study by Kramers et al. [22] reported increased gastrointestinal symptoms with metformin, although most were mild. In the TAME-PKD trial [8], dose reduction was required in over half of metformin-treated participants; however, treatment discontinuation was uncommon, and gastrointestinal quality-of-life scores remained stable. Importantly, hypoglycemia risk was not increased, and no serious adverse events such as lactic acidosis were observed, supporting the safety of metformin in non-diabetic individuals with preserved kidney function [28].

The ongoing IMPEDE-PKD trial (NCT04939935), a large multinational RCT comparing metformin with placebo enrolling 1,174 participants, is designed with a larger sample size and longer follow-up periods and is expected to provide more definitive evidence on the efficacy and safety of metformin in ADPKD [29]. The findings of this trial will be important to build on the trends observed in our meta-analysis and to further clarify the therapeutic role of metformin in this population.

A potential additive or synergistic role of metformin with tolvaptan has been hypothesized, as the two drugs act through distinct but complementary mechanisms in cystogenesis. Tolvaptan reduces cAMP levels, thereby limiting cyst fluid secretion, while metformin activates AMPK, inhibiting proliferative and secretory pathways such as mTOR and CFTR [30, 31]. In Kramers et al. [22] concomitant use of tolvaptan and metformin did not demonstrate additional benefit, and a secondary analysis of the TEMPO 3:4 and REPRISE trials likewise found no clear additive effect, though the combination appeared safe and well tolerated [32]. Evidence is therefore insufficient to define whether metformin provides complementary efficacy to tolvaptan, and further studies specifically designed to test this strategy are warranted.

An important gap in the current literature is the lack of studies that stratify patients by risk of progression (e.g., Mayo Clinic classification), genetic mutations (PKD1 vs. PKD2), age, sex, and the presence of metabolic comorbidities, all of which may critically influence therapeutic response. Similarly, no clinical outcome data stratified by PKD1 or PKD2 mutation status, age or comorbidity were available across the included studies. Details of Mayo Imaging Classification and PKD genotype distribution in each trial are summarized in Supplementary Table 3. Future trials that incorporate these variables will be essential to identify subgroups most likely to benefit from metformin and to advance a more individualized approach to ADPKD management.

This systematic review and meta-analysis aimed to repurpose metformin as a potential disease-modifying therapy in non-diabetic patients with ADPKD and its findings are clinically meaningful given the scarcity of pharmacologic options for ADPKD. While tolvaptan remains the only approved disease-modifying treatment, its use is limited by cost, hepatotoxicity risk, and aquaretic side effects [30]. In contrast, metformin is widely accessible, inexpensive, and has a long-established safety profile in other populations [10, 16, 33, 34] Thereby, even a modest improvement in kidney function could make metformin a valuable adjunct therapy in carefully selected patients with ADPKD [22, 32].

### Limitations

Some limitations of our study should be acknowledged. The small number of included studies and patients limits statistical power and generalizability. The relatively short follow-up durations in several trials may also have been insufficient to capture long-term treatment effects. In addition, the representativeness of the available evidence is limited, as only one trial was conducted in an Indian population, with the remainder based in Europe or the United States. Risk of bias was rated as ‘some concerns’ in at least two domains for certain studies, including bias in outcome measurement and overall risk of bias.

Furthermore, heterogeneity in metformin dosing and baseline disease severity may have introduced variability in treatment effects and limited the generalizability of our findings. In addition, insufficient data across the included trials precluded meaningful subgroup analyses by age, BMI, sex, comorbidities, Mayo class, or genetic subtype. While several trials included a high proportion of patients classified as Mayo 1C–1D, the Venkatasubramanian study [24] specifically excluded those at highest risk (1D–1E) and primarily enrolled lower-risk patients. The inclusion of slowly progressive patients (Mayo 1A–1B) may have further diluted any potential treatment effect, whereas rapid progressors (Mayo 1C–1E) are the subgroup most clinically relevant for evaluating disease-modifying therapies. Finally, none of the included studies assessed hard clinical endpoints such as progression to kidney failure or the need for kidney replacement therapy, further limiting clinical extrapolation.

Taken altogether these limitations highlight the need for large, well-designed, parallel-arm randomized controlled trials with standardized outcome definitions, diverse study populations, and extended follow-up to confirm the potential benefits and define the optimal role of metformin in the management of non-diabetic ADPKD.

## Conclusion

This meta-analysis of four RCTs, including 213 non-diabetic patients with ADPKD, revealed no significant effect of metformin on slowing the decline in the eGFR or on limiting htTKV. Gastrointestinal adverse events were more common in the metformin group, though generally mild, and no increased risk of hypoglycemia was observed, supporting a favorable safety profile in patients with preserved kidney function. However, the relatively short and inconsistent follow-up durations across trials, which are not well matched to the chronic course of ADPKD, limit the ability to assess long-term outcomes such as the annual decline in eGFR, long-term htTKV growth, and risk of progression to kidney failure. Larger, well-powered studies with extended follow-up are needed to better define the potential therapeutic role of metformin in non-diabetic patients with ADPKD.

## Electronic supplementary material

Below is the link to the electronic supplementary material.

Supplementary Material 1

## Data Availability

All data generated or analyzed during this study are included in this published article and its supplementary material files.

## Abbreviations

2DG: 2-Deoxy-D-Glucose
ADPKD: Autosomal Dominant Polycystic Kidney Disease
AMPK: AMP-activated Protein Kinase
AMP/ATP: Adenosine Monophosphate/Adenosine Triphosphate
CFTR: Cystic Fibrosis Transmembrane Conductance Regulator
CKD: Chronic Kidney Disease
CI: Confidence Interval
df: Degrees of Freedom
eGFR: Estimated Glomerular Filtration Rate
EMT: Epithelial-Mesenchymal Transition
EMA: European Medicines Agency
FDA: Food and Drug Administration
GLMM: Generalized Linear Mixed-effects Model
GI: Gastrointestinal
GRADE: Grading of Recommendations Assessment, Development and Evaluation
GFR: Glomerular Filtration Rate
htTKV: Height-adjusted Total Kidney Volume
IV: Inverse Variance
KDIGO: Kidney Disease: Improving Global Outcomes
MD: Mean Difference
mTOR: Mechanistic Target of Rapamycin
mTORC1: Mechanistic Target of Rapamycin Complex 1
PFT: Freeman-Tukey Double Arcsine Transformation
PLOGIT: Logit Transformation for Proportions
PRISMA: Preferred Reporting Items for Systematic Reviews and Meta-Analyses
PROSPERO: International Prospective Register of Systematic Reviews
RCT: Randomized Controlled Trial
REML: Restricted Maximum Likelihood
RoB-2: Risk of Bias 2 Tool
RR: Risk Ratio
SMD: Standardized Mean Difference
TGF-β: Transforming Growth Factor Beta
