# Supplementary material for: Metformin in non-diabetic patients with autosomal dominant polycystic kidney disease: a systematic review and meta-analysis of randomized controlled trials
Source: BMC Nephrol. 2025 Nov 18;26:646. doi: 10.1186/s12882-025-04575-5 (PMC12625526; doi:10.1186/s12882-025-04575-5)
Supplement: Supplementary file 1 — Supplementary Material 1 [file 12882_2025_4575_MOESM1_ESM.docx]

**Supplementary Material**

**Metformin in Non-diabetic Patients with Autosomal Dominant Polycystic Kidney Disease: A Systematic Review and Meta-Analysis of Randomized Controlled Trials**

Vitor Almeida,^1^ Lucas Maciel,^2^ MD, Ana Ramos,^3^ Carla Sousa,^4^ Maria Ferraz,^5^ Luisalice Afonso,^6^ MD, Paula Dibo^7^ MD, PhD, Ivana Nunes,^8^ MD, PhD.

^1^ Federal University of Catalão, Goiás, Brazil

^2^ General Hospital of Goiânia, Goiás, Brazil

^3^ Pontifical Catholic University of Paraná, Paraná, Brazil

^4^ Jean Piaget University at Angola, Luanda, Angola

^5^ Vila Velha University, Espírito Santo, Brazil

^6^ Federal University of Cariri, Ceará, Brazil

^7^ Division of General Internal Medicine, Department of Medicine, Emory University, Atlanta, GA, USA

^8^ Division of Nephrology, Department of Internal Medicine, General Hospital of Goiânia, Goiás, Brazil

**Table of contents**

Full search strategy……………………………………….…………………………...2

Reasons for exclusion after full-text review..…………………………………...…..3

Supplementary Table 1………....………………………………………………….…4

Supplementary Table 2………....………………………………………………….…6

Supplementary Table 3………....………………………………………………….…6

Supplementary Figures…………..……………………………………………………7

**Full search string**

● PubMed

("Polycystic Kidney, Autosomal Dominant"[Mesh] OR "Autosomal Dominant Polycystic Kidney" OR "Adult Polycystic Kidney Disease" OR ADPKD OR "polycystic kidney disease") AND ("Metformin"[Mesh] OR Metformin OR Glucophage OR dimethylbiguanide)

● Embase

('kidney polycystic disease'/exp OR 'autosomal dominant polycystic kidney' OR 'adult polycystic kidney disease' OR 'adpkd' OR 'polycystic kidney disease') AND ('metformin'/exp OR 'metformin' OR 'glucophage' OR 'dimethylbiguanide')

● Cochrane Library

("Polycystic Kidney Autosomal Dominant" OR "Autosomal Dominant Polycystic Kidney" OR "Adult Polycystic Kidney Disease" OR ADPKD OR "polycystic kidney disease") AND (Metformin OR Glucophage OR dimethylbiguanide)

**Reasons for exclusion after full-text review**

● Protocol studies

Seliger S L, Abebe K Z, Hallows K R, Miskulin D C, Perrone R D, Watnick T, Bae K T. A randomized clinical trial of metformin to treat autosomal dominant polycystic kidney disease. J Am Soc Nephrol. 2024;35(5):879-888. doi:10.1681/ASN.2023060666

Metformin as a novel therapy for autosomal dominant polycystic kidney disease. ClinicalTrials.gov Identifier: NCT02656017. Registered 2015. Updated May 31, 2018.

Feasibility study of metformin therapy in ADPKD. ClinicalTrials.gov Identifier: NCT02903511. Registered 2016. Updated May 31, 2018.

Metformin vs tolvaptan for treatment of autosomal dominant polycystic kidney disease. ClinicalTrials.gov Identifier: NCT03764605. Registered 2018. Updated March 31, 2019.

Implementation of Metformin theraPy to Ease Decline of Kidney Function in Polycystic Kidney Disease (IMPEDE-PKD). ClinicalTrials.gov Identifier: NCT04939935. Registered 2021. Updated July 31, 2021.

Evaluation of metformin and tolvaptan in slowing disease progression of autosomal dominant polycystic kidney disease (ADPKD). EU Clinical Trials Register Identifier: EUCTR2018-000477-77-IT. Registered 2020. Updated March 31, 2021.

Comparison of metformin therapy with placebo therapy on progression of disease in the patients with autosomal dominant polycystic kidney disease. Clinical Trials Registry - India Identifier: CTRI/2024/06/069541. Registered 2024. Updated August 31, 2024.

● Wrong study design

Pisani A, Riccio E, Bruzzese D, Sabbatini M. Metformin in autosomal dominant polycystic kidney disease: experimental hypothesis or clinical fact? J Nephrol. 2017;30(3):339-347. doi:10.1007/s40620-016-0306-2

● No outcomes of interest for non-diabetic patients with ADPKD

Kuo I‑C, Lin M‑Y, Tsao Y‑H, Chiu Y‑W, Lee J‑J. Metformin use and clinical outcomes in autosomal dominant polycystic kidney disease: a nationwide cohort study. *Biomedicines.* 2025;13(3):635. doi: 10.3390/biomedicines13030635

Mahendran R, Lim S K, Ong K C, Chua K H, Chai H C. Natural-derived compounds and their mechanisms in potential autosomal dominant polycystic kidney disease (ADPKD) treatment. Front Pharmacol. 2021;12:682620. doi:10.3389/fphar.2021.682620

Okyere P, Ephraim R K D, Okyere I, Attakorah J, Serwaa D, Essuman G, Abaka-Yawson A, Adoba P. Demographic, diagnostic and therapeutic characteristics of autosomal dominant polycystic kidney disease in Ghana. BMC Nephrol. 2024;25(1):145. doi:10.1186/s12882-024-02989-4

● No control group

Sorohan BM, Ismail G, Andronesi A, Micu G, Obrișcă B, Jurubiță R, Sinescu I, Baston C. A single-arm pilot study of metformin in patients with autosomal dominant polycystic kidney disease.*BMC Nephrology.* 2019;20(1):276. doi: 10.1186/s12882-019-1463-2

Stanley IK, Palma AM, Viecelli AK, Orellana JM, Harris DC, Gorriz JL, Jardine MJ, Pei Y, Chapman AB. A secondary analysis of concurrent use of metformin and tolvaptan in ADPKD tolvaptan trials. *Journal of Nephrology.* 2024;37(6):1417–1419. doi: 10.1007/s40620-024-01906-x

Wang W, You Z, Steele CN, et al. Changes in tubular biomarkers with dietary intervention and metformin in patients with autosomal dominant polycystic kidney disease: a post‑hoc analysis of two clinical trials. *BMC Nephrology.* 2024;25:206. doi: 10.1186/s12882-024-03643-6

● Conference abstracts:

Steele C, Klawitter J, Wang W, You Z, Catenacci V, Struemph T, George D, Gitomer B Y, Brosnahan G M, Chonchol M, Nowak K L. Metabolomic changes over 1 year following drug or lifestyle interventions in autosomal dominant polycystic kidney disease (ADPKD). *J Am Soc Nephrol.* 2021;32(10 Suppl):405. doi: 10.1681/ASN.20213210S1405a

Datta A, Ray Chaudhury A, Sircar D, Sadhukhan S, Pal A, Bhattacharjee K, Dasgupta S. Role of metformin in ADPKD: experience from a tertiary care centre in Eastern India. *Kidney Int Rep.* 2025;10(2 Suppl):S632–S633. doi: 10.1016/j.ekir.2024.11.1126

Datta A, Ray Chaudhury A, Kar S, Pal A, Bhattacharjee K, Sen D. A single center experience of metformin therapy in retarding progression of ADPKD. *Kidney Int Rep.* 2023;8(3 Suppl): S278–S279. doi: 10.1016/j.ekir.2023.02.627

**Supplementary Table 1.** Risk of bias summary for randomized studies (RoB 2)


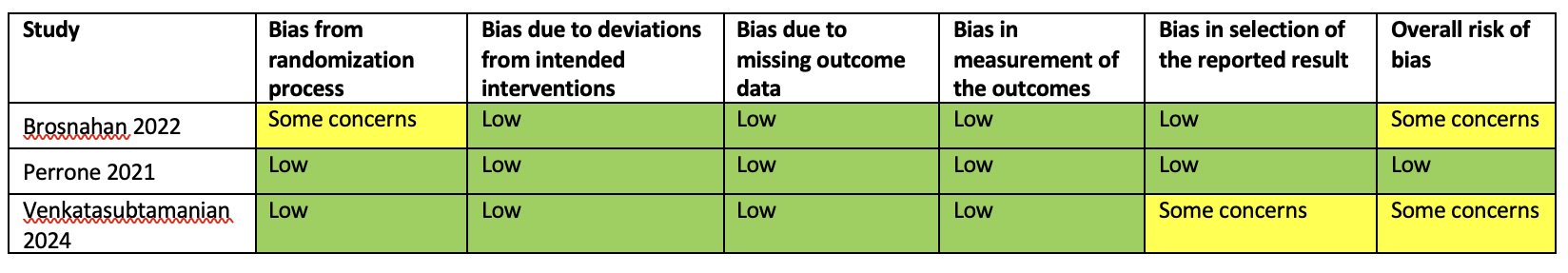


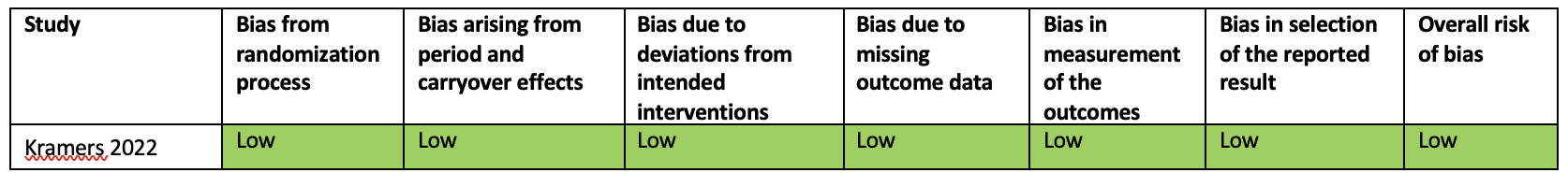


Sterne JAC, Savović J, Page MJ, Elbers RG, Blencowe NS, Boutron I, Cates CJ, Cheng H-Y, Corbett MS, Eldridge SM, Hernán MA, Hopewell S, Hróbjartsson A, Junqueira DR, Jüni P, Kirkham JJ, Lasserson T, Li T, McAleenan A, Reeves BC, Shepperd S, Shrier I, Stewart LA, Tilling K, White IR, Whiting PF, Higgins JPT. RoB 2: a revised tool for assessing risk of bias in randomised trials. *BMJ* 2019; **366**: l4898

**Supplementary Table 2.** Grading of Recommendations Assessment, Development and Evaluation (GRADE)

**
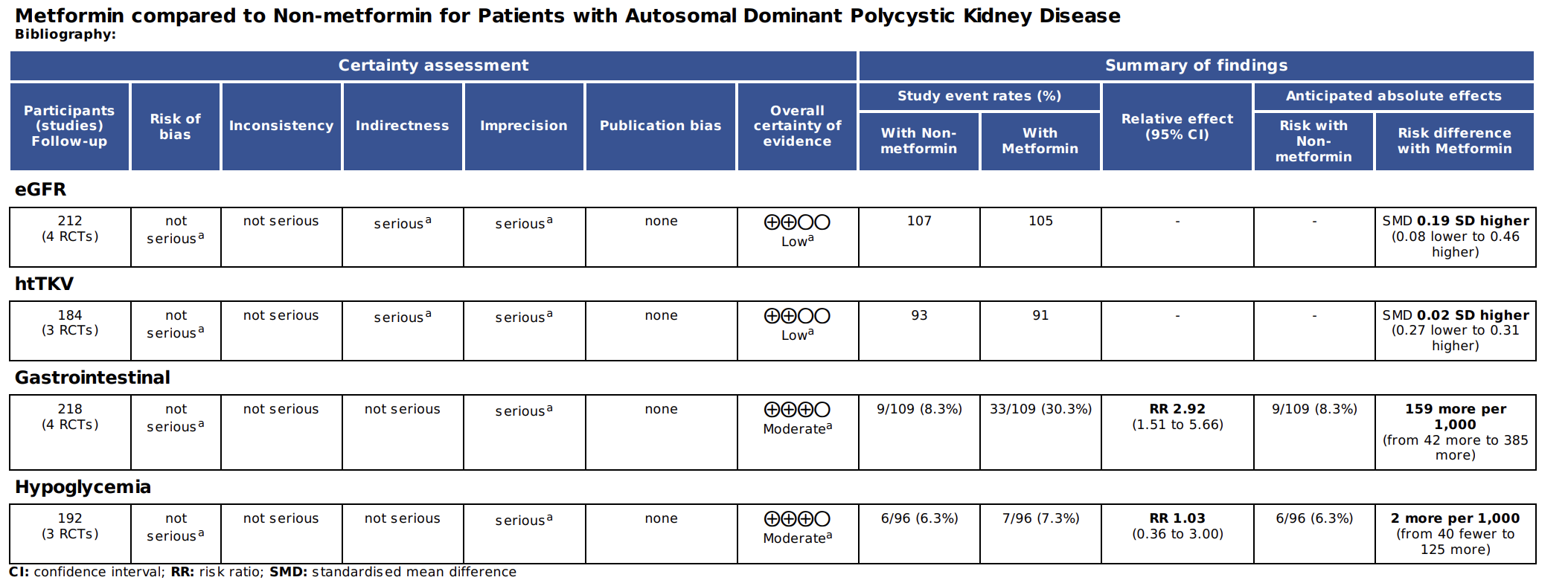
**

Explanations

a. Limited number of trials, few events, and short follow-up.

**Supplementary Table 3.** Extended Table of PKD Genotype and Mayo Classification of included studies

| Study, years | Mayo Class  1A, no.  M/C | Mayo Class  1B, no.  M/C | Mayo Class  1C, no.  M/C | Mayo Class  1D, no.  M/C | Mayo Class  1E, no. M/C | PKD1 genotype, no. (%) M/C | PKD2 genotype, no. (%) M/C |
| --- | --- | --- | --- | --- | --- | --- | --- |
| Brosnahan, 2022 | 1/2 | 6/7 | 8/14 | 7/1 | 4/1 | NR | NR |
| Kramers, 2022 | 4/4* | 4/4* | 9/9* | 9/9* | 9/9* | NR | NR |
| Perrone, 2021 | 9/6 | 13/15 | 14/12 | 6/6 | 4/4 | 37/28 | 7/10 |
| Venkatasubramania, 2024 | 10/10 | 10/12 | 5/4 | 1/0 | NR | 4/2 | 0/1 |

M: metformin users; C: Control group; PKD: Polycystic Kidney Disease; NA: Not Reported; * Mayo Class MIC 1A and 1B were combined into a single composite group, as were Mayo Class MIC 1C, 1D; and 1E.

**Supplementary Figures**

**
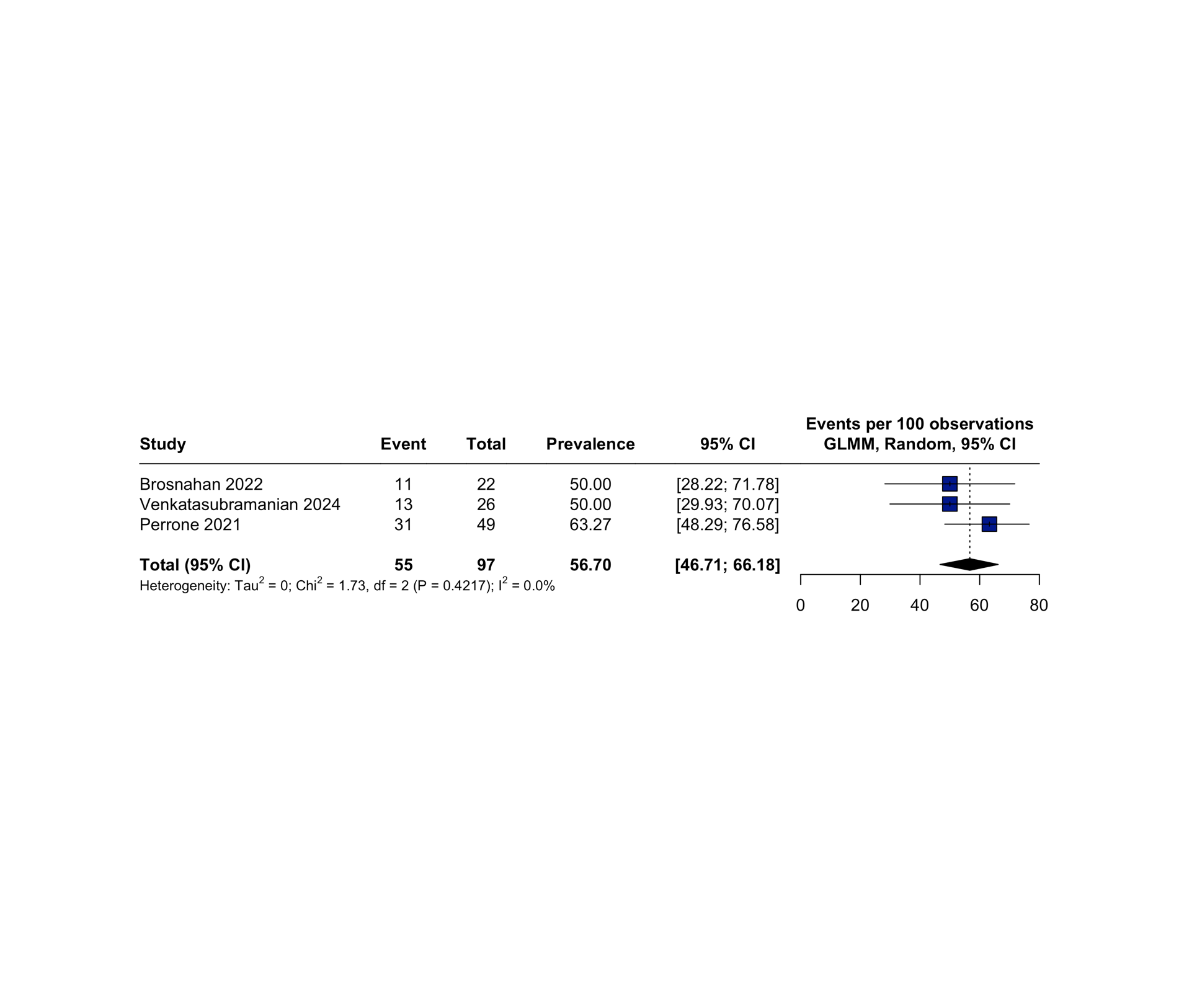
**

**Supplementary Figure 1.** Subgroup analysis of tolerability in the metformin arm, excluding data from Kramer's 2022 study **
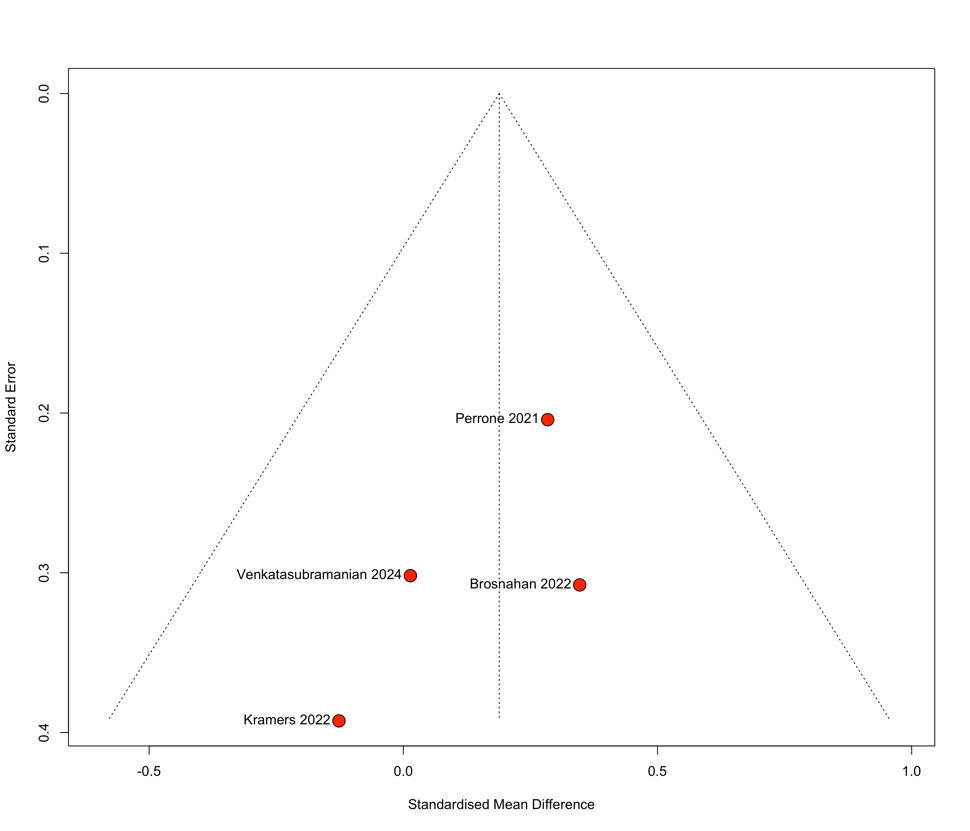
**

**Supplementary Figure 2A.** Funnel plot showing the outcome of kidney function decline rate.


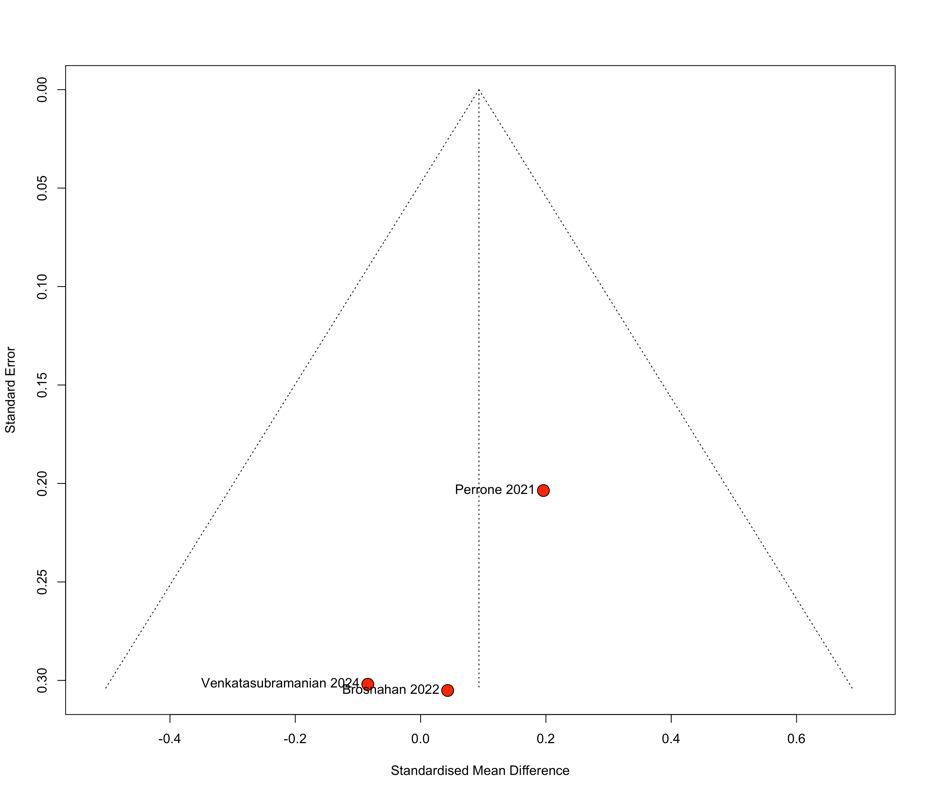


**Supplementary Figure 2B.** Funnel plot showing the outcome of height-adjusted total kidney volume (htTKV).


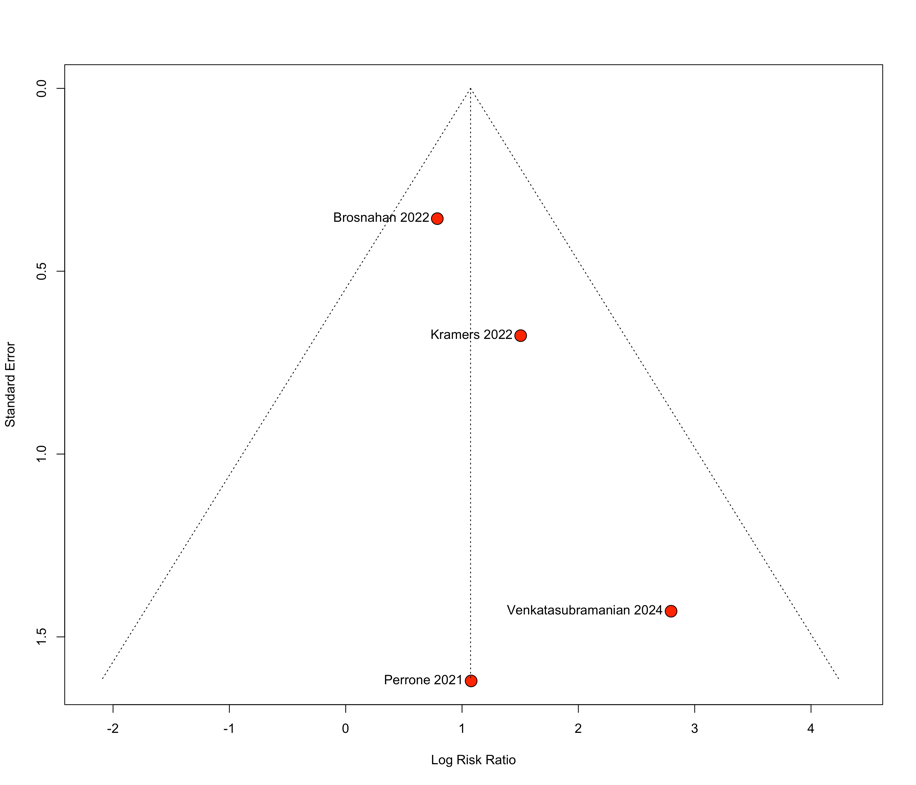


**Supplementary Figure 2C.** Funnel plot showing the outcome of gastrointestinal adverse events.


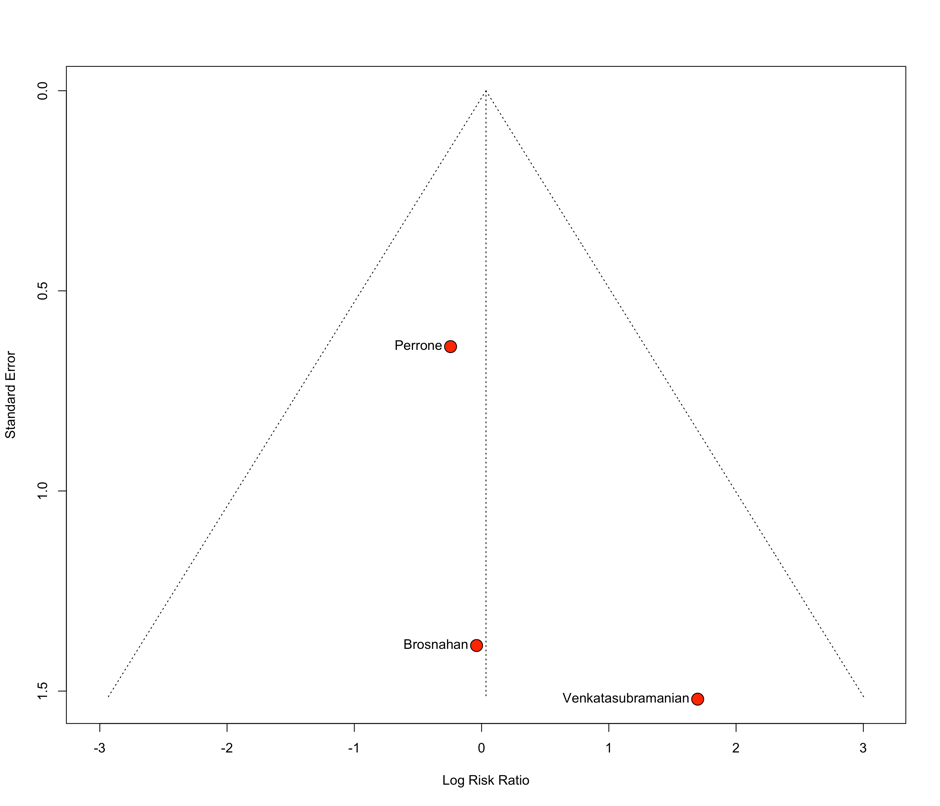


**Supplementary Figure 2D.** Funnel plot showing the outcome of hypoglycemia.


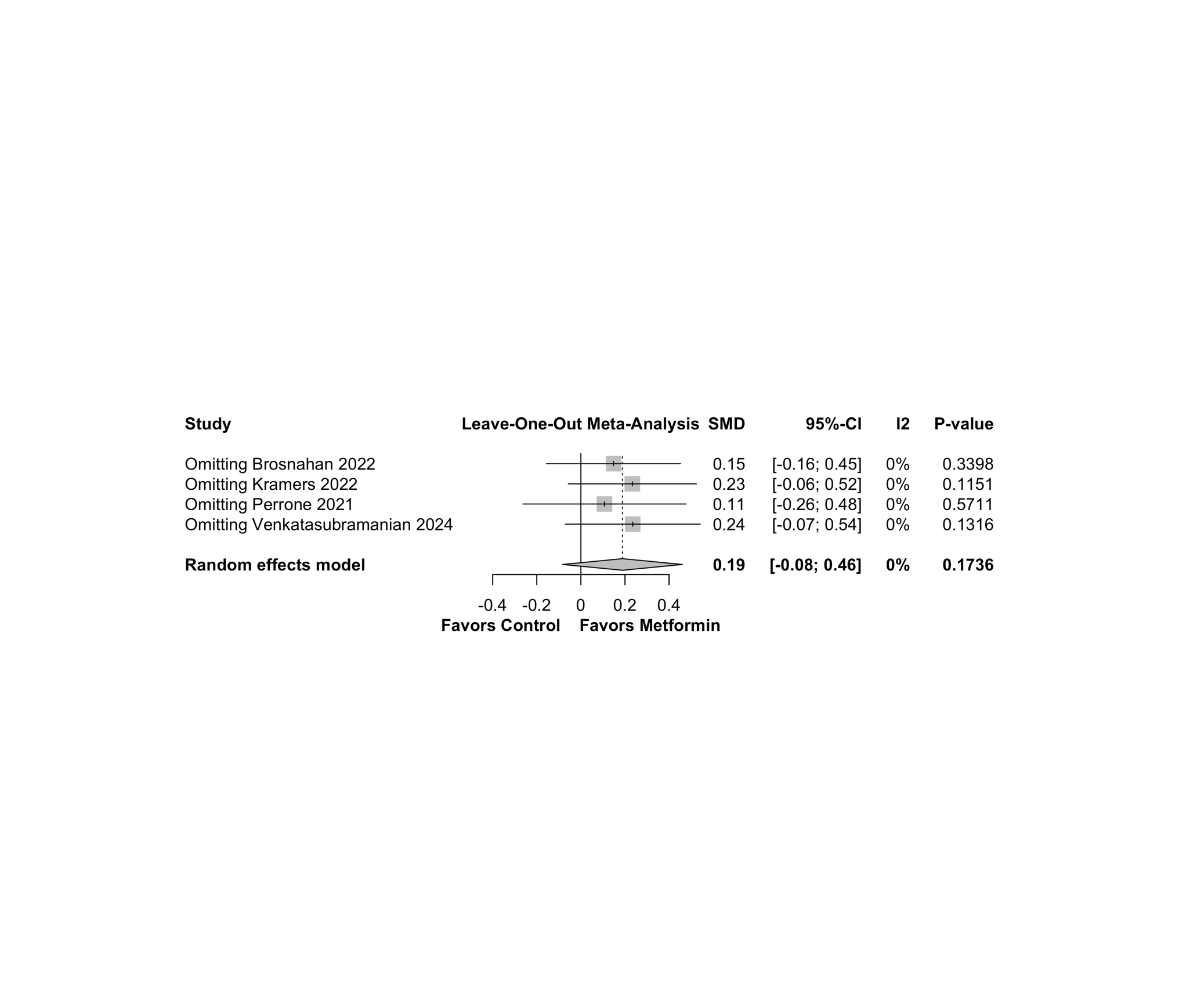


**Supplementary Figure 3A.** Leave-one-out sensitivity analysis assessing the outcome of kidney function decline rate


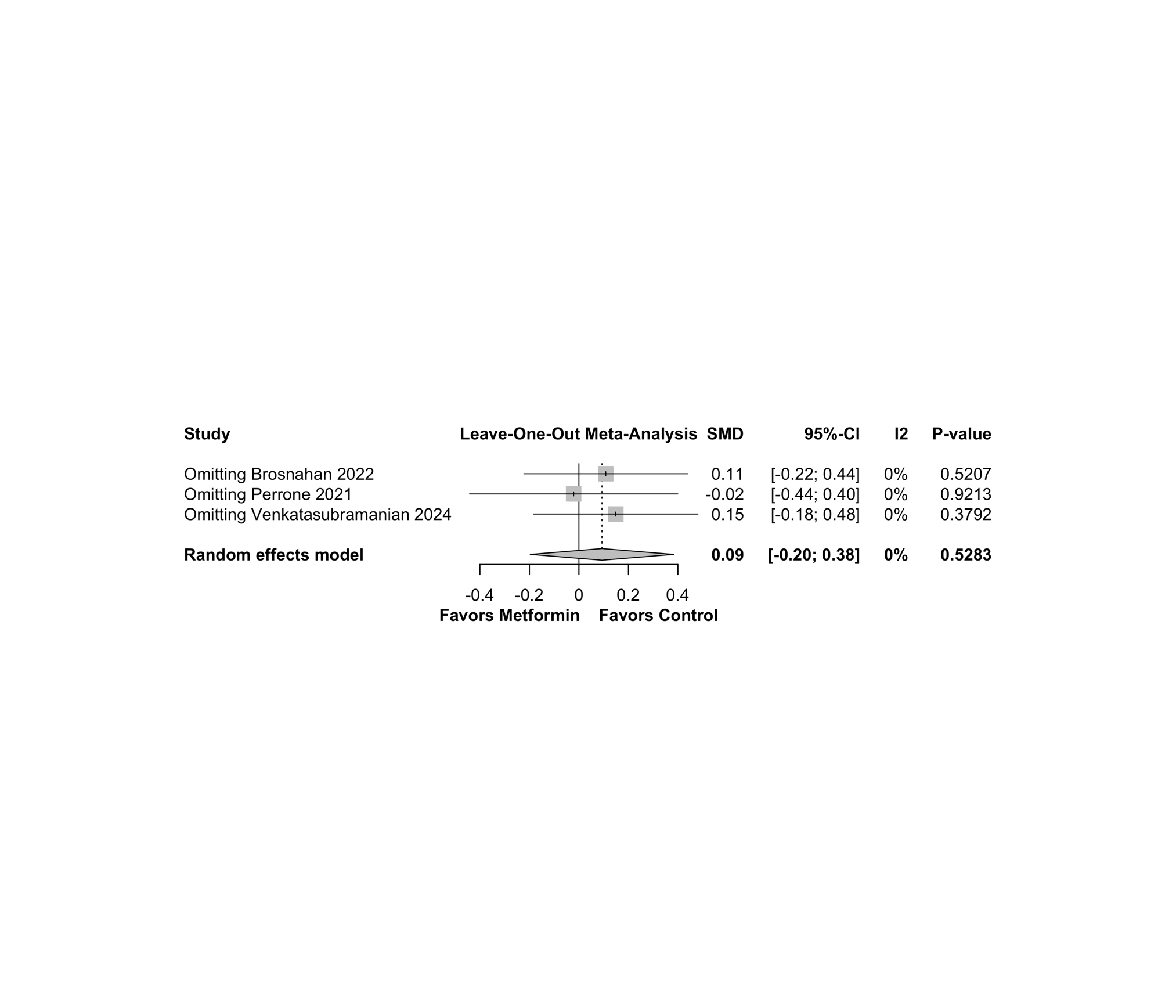


**Supplementary Figure 3B.** Leave-one-out sensitivity analysis assessing the outcome of height-adjusted total kidney volume (htTKV).

**
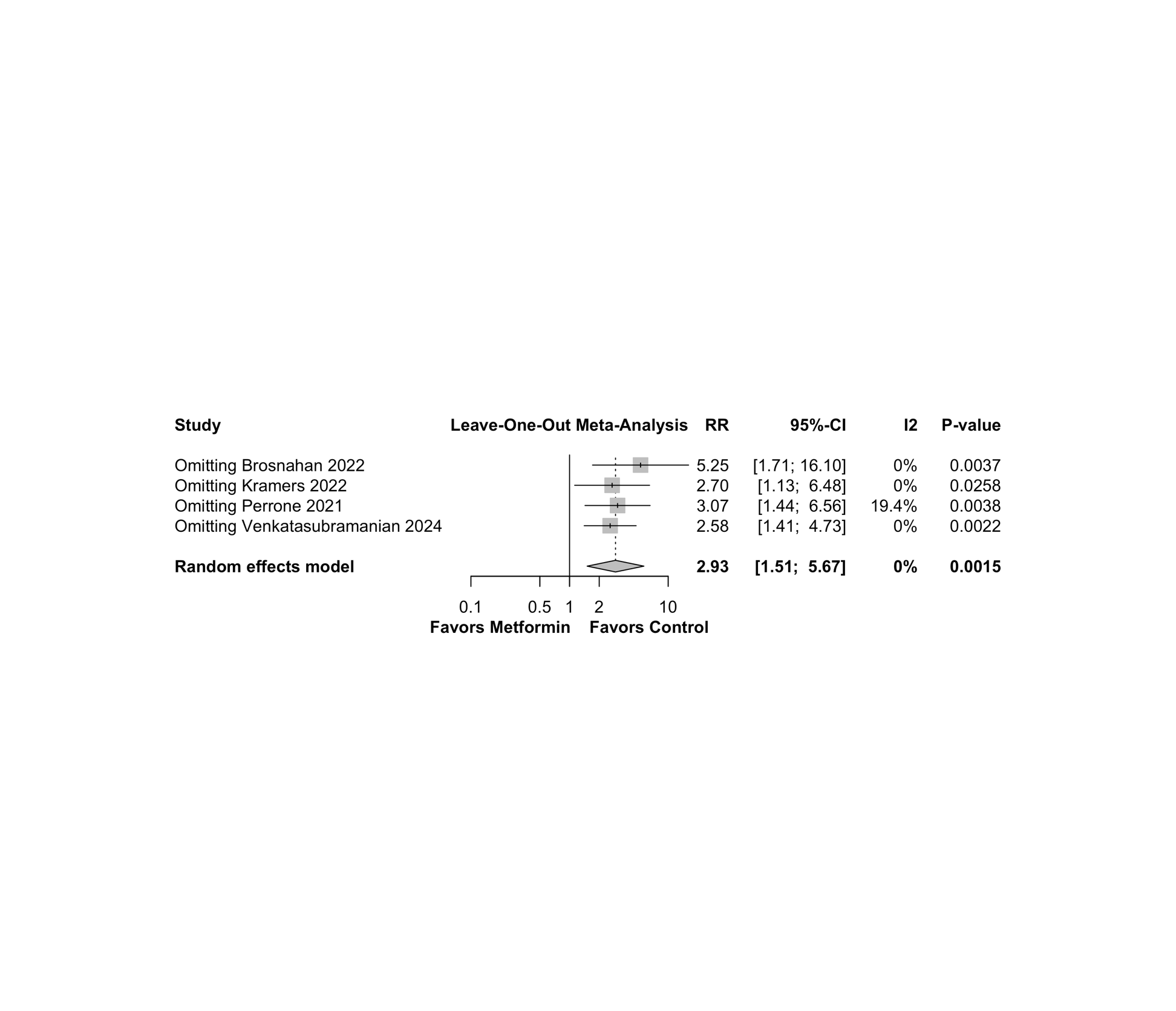
**

**Supplementary Figure 3C.** Leave-one-out sensitivity analysis assessing the outcome of gastrointestinal adverse events


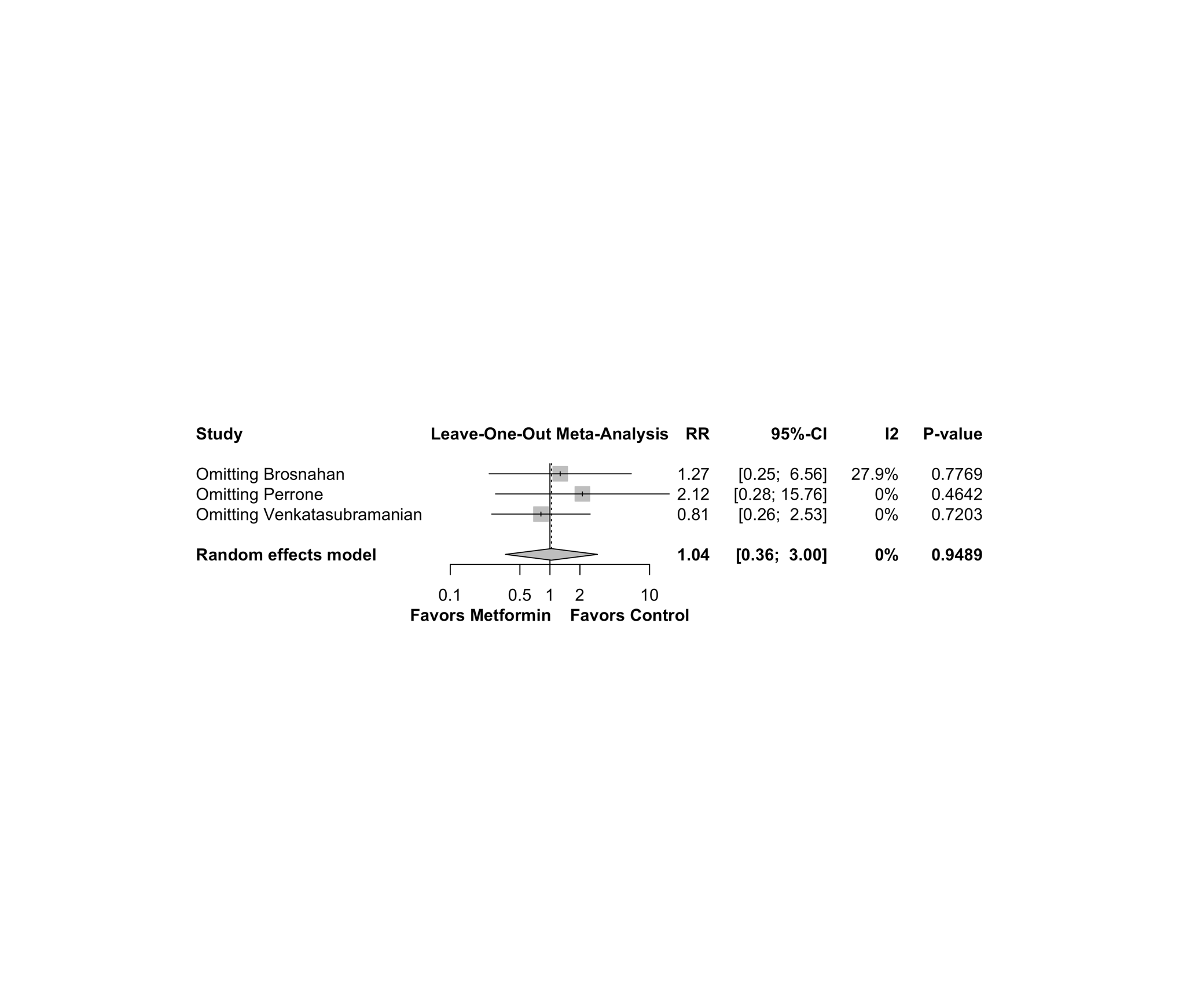


**Supplementary Figure 3D.** Leave-one-out sensitivity analysis assessing the outcome of hypoglycemia.


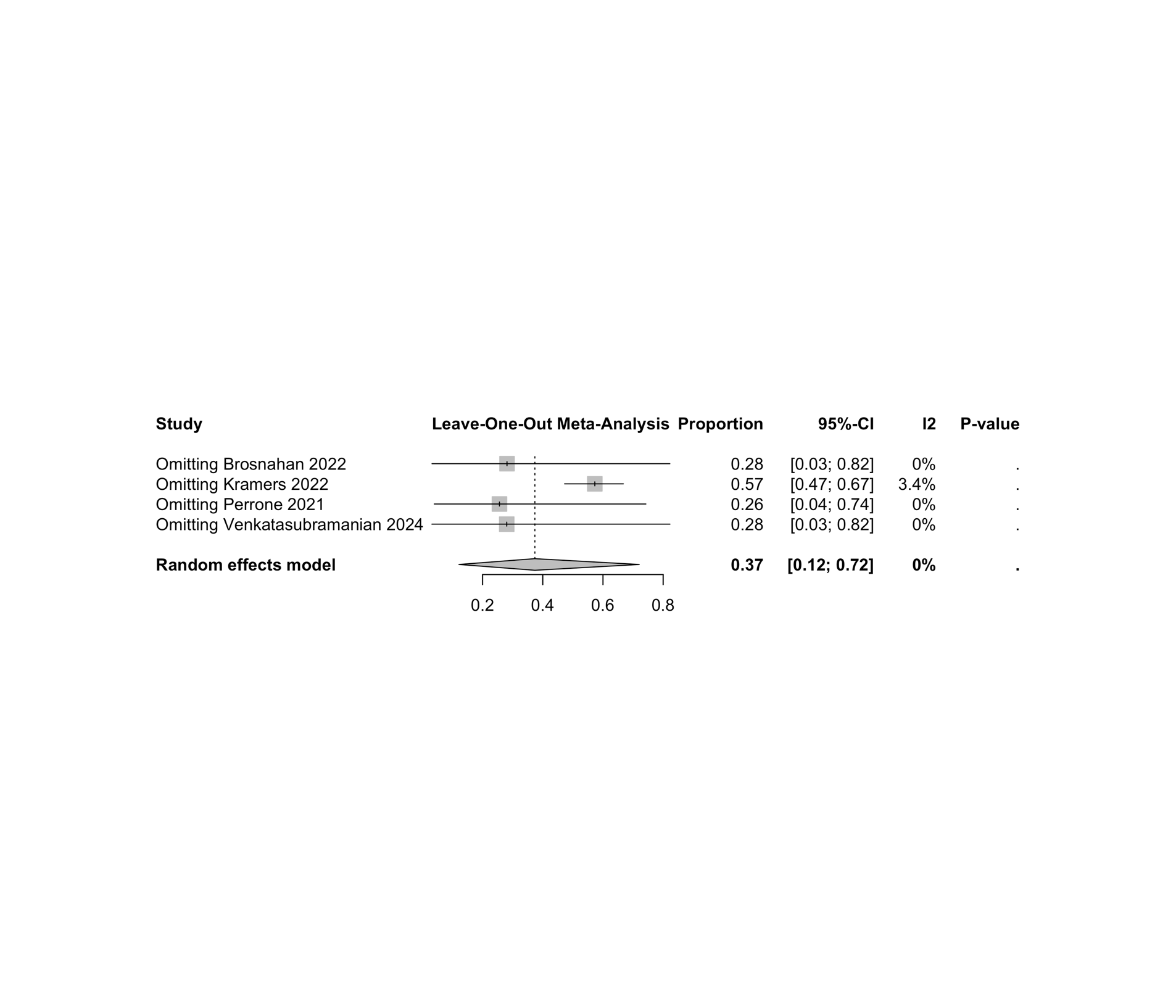


**Supplementary Figure 3E.** Leave-one-out sensitivity analysis assessing the outcome of tolerability defined as dose reduction or discontinuation of metformin.

**
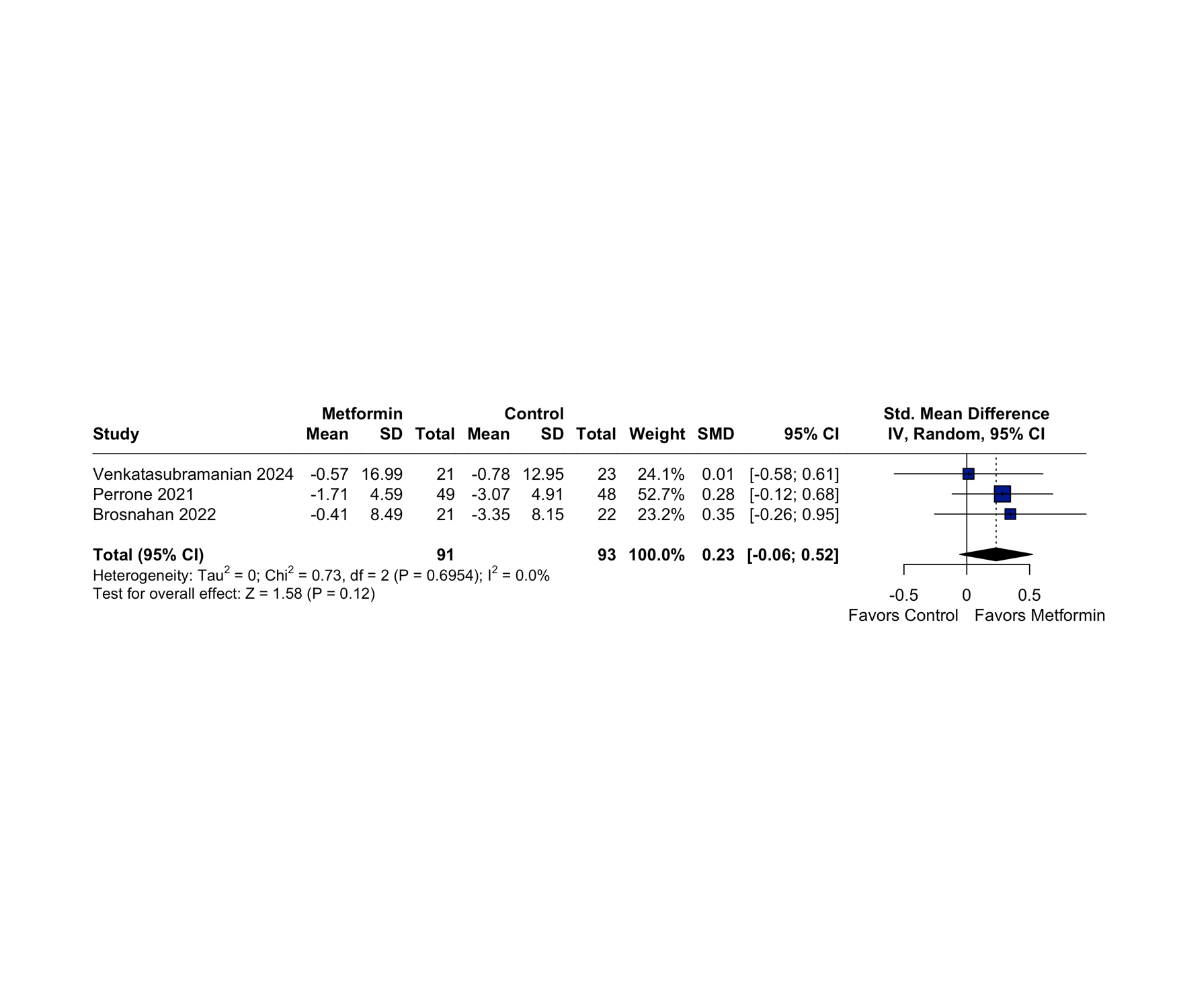
**

**Supplementary Figure 4.** Forest plot for kidney function rate decline without Kramers 2022.
